# Soft drink prices, sales, body mass index and diabetes: Evidence from a panel of low-, middle- and high-income countries

**DOI:** 10.1016/j.foodpol.2017.09.002

**Published:** 2017-12

**Authors:** Yevgeniy Goryakin, Pablo Monsivais, Marc Suhrcke

**Affiliations:** a Health Economics Group, Norwich Medical School, University of East Anglia, Norwich NR4 7TJ, UK; b UKCRC Centre for Diet and Activity Research, School of Clinical Medicine, University of Cambridge, Cambridge CB2 0QQ, UK; c Centre for Health Economics, University of York, York YO10 5DD, UK; d Department of Nutrition and Exercise Physiology, Elson S Floyd College of Medicine, Washington State University, Spokane, WA 99210-1495, USA; e Luxembourg Institute for Socio-Economic Research, L-4366 Esch-sur-Alzette/Belval, Luxembourg

**Keywords:** Soft drink prices, Diabetes, Body mass index, Fiscal policies

## Abstract

We take advantage of four different cross-country datasets containing data on 78 countries for the period 1999–2014, in order to assess the relationship of carbonated soft drinks’ sales, as well as their prices, with body mass index (BMI), overweight, obesity and diabetes. Using an ecological study design and multivariate regression longitudinal estimation approaches, we find that carbonated soft drink sales were significantly positively related to BMI, overweight and obesity – but only in the low and lower-middle income countries. This finding was robust to a number of sensitivity and falsification checks. In this sub-sample, an increase in per capita soft drink sales by 1 litre per year was related to an increase of BMI by about 0.009 kg/m^2^ (p < 0.1).. This is a small effect, implying that halving annual consumption per capita in this group of countries would result in a drop of BMI by only about 0.03 kg/m^2^. Although soft drink prices were negatively related to weight-related outcomes in the sample of higher middle income and high income countries, this finding was not robust to falsification checks. The results thus suggest that sales restrictions to steer consumers away from soft drinks could indeed have a beneficial health effects in poorer countries, although the effect magnitude appears to be very small. However, given potential limitations of using ecological research design, results from individual level studies would be required to further ascertain the role of soft drink sales and prices in obesity and diabetes.

## Highlights

• We use global cross-country panel data on soft drink prices, sales and health.
• We assess the effect of relative soft drink prices and sales on health outcomes.
• Soft drink sales are significantly positively related to BMI and to diabetes among the low and lower-middle income countries.
• The expected inverse relationship between relative soft drink prices and weight-related turned out as not robust.

## 1 Introduction

Consumption of soft drinks, and in particular of sugar-sweetened beverages (SSBs), has been singled out as a global public health concern (Vartanian et al., 2007), in light of their contribution to total dietary sugar intake, high glycemic index and purported role in excess energy intake (Vartanian et al., 2007). Soft drinks consumption has been growing globally (Popkin, 2010): as (Moodie et al., 2013) have shown, the average annual growth rate of per capita soft drink consumption between 1997 and 2009 was 5.2% in low and middle income countries, and 2.4% in high income countries.

Converging lines of evidence indicate that SSBs are associated with greater adiposity and weight gain. Three systematic reviews (Malik et al., 2013, Malik et al., 2006, Vartanian et al., 2007), as well as another review article (Hu, 2013) concluded there was evidence of a positive association between individual-level soft drink intake and body weight or the odds of overweight and obesity. Interestingly, larger effect sizes were observed in experimental than in observational studies, suggesting a potential attenuation bias in non-experimental studies. Likewise, estimates were found to be larger in non-industry-sponsored studies. More recent trials provided yet stronger causal evidence, indicating that replacement of SSBs with non-caloric beverages reduced weight gain and fat accumulation in normal-weight children (de Ruyter et al., 2012)

Consumption of SSBs may also increase the risk of type-2 diabetes. For example, ecological studies have suggested correlations between increasing consumption of soft drinks and rates of diabetes (Basu et al., 2013, Greenwood et al., 2014, Gross et al., 2004, Schulze et al., 2004). In their meta-analysis of 11 studies, Malik et al. (2010) estimated that people in the highest quintile of SSB consumption have an about 26% greater risk of diabetes compared to people in the lowest one. Nevertheless, it should be emphasized that the difference in these consumption thresholds is very large, with people in the first group having 1–2 servings of SSBs a day, and those in the latter having none or one serving a month. Finally, in another recent systematic review, one extra serving per day of SSB was found to be related to an about 18% greater risk of diabetes, with nearly nine percent of USA type-2 diabetes cases attributable to SSBs (Imamura et al., 2015).

From the policy point of view, it is important to know how overweight/obesity and diabetes are related to consumption and prices of soft drinks and juices. For the association to hold there should be a correlation not only between consumption of these drinks and overweight/diabetes, but also between prices and consumption. While a recent meta-analysis estimated a rather large combined own price elasticity of SSBs (Cabrera Escobar et al., 2013) of around −1.3, this finding was based mostly on studies from high income countries (8 out of 10). Moreover, the study with the largest elasticity (-4.45) included in the *meta*-analysis was actually restricted to children and adolescents only. Yet, if there was any negative effect of prices on average BMI, it was not possible to conclude this from the studies reviewed: in 2 out of 5 the association was positive, while in the remaining 3 the association appeared to be small. Nevertheless, the association between prices and overweight/obesity was in the expected negative direction in 8 studies reviewed, although it appears it was significant in only two of them. Similarly, Schroeter et al. (2008) concluded that even in the US, only modest changes in population weight will result from increasing soft drinks taxation, because their consumption represents only 7% of total energy intake. In a recent modelling study for the UK, Briggs et al. (2013) also estimated a rather small effect, implying that an increase in the tax on SSD by 20% would lead to a 1.3% reduction in the proportion of people who are overweight.

In sum, the existing evidence on the effect of soft drink consumption on BMI/overweight/diabetes, as well as on the effect of prices on BMI/overweight/diabetes, is incomplete with most evidence coming from higher-income countries. The evidence base would benefit from more research based on longitudinal and/or individual-level data (Basu et al., 2013). In this paper, we compose and utilize a large, cross-country longitudinal dataset, attempting to find out how the soft drinks sales per capita, as well as their prices, are related to average BMI, overweight, obesity and diabetes prevalence in a sample of 78 low, middle and high income countries.

## 2 Methods

### 2.1 Data

#### 2.1.1 Outcome variables

The outcome data in our paper is taken from several sources. First, the data on average, age-standardized country-level BMI levels, overweight, obesity and diabetes prevalence are from the NCD Risk Factor Collaboration (NCD-RisC)^1^ project, available annually between 1999–2014. The data have been estimated on the basis of a large number of surveys, articles and epidemiological studies (Danaei et al., 2011a, Danaei et al., 2011b, Finucane et al., 2011a).

#### 2.1.2 Key independent variables

The data on carbonated soft drink sales^2^ and prices is from the Euromonitor, Passport Global Market Information Database (2014 edition). The data contains information on carbonated soft drink sales and prices from 78 countries world-wide, spanning the period from 1999 to 2014. We obtained carbonated soft drink prices (in US$ per litre, historic constant 2013 prices) by dividing the total off-trade value of carbonated soft drinks by the off-trade volume of these beverages (in million litres). To facilitate inter-country comparisons, we divided these prices by the price level index (PLI) produced by the World Bank. PLI is a ratio of purchasing power parities to corresponding exchange rates, and it is often used to compare prices between different countries^3^. Per capita sales of carbonated soft drinks were derived by dividing the off-trade volume of these drinks by the population of each country. To reduce potential for reverse causality, the main independent covariates of interest (prices as well as sales of soft drinks) were lagged by one year in all models.

#### 2.1.3 Control variables

When estimating the association between carbonated soft drink sales and weight-related outcomes, it is important to keep in mind that soft drink production is highly globalized, with more than half of it being controlled by large international corporations, such as Coca-Cola and PepsiCo (Moodie et al., 2013). Therefore, we control for the degree of globalization as measured by the KOF Globalization Index (Dreher, 2006b), which was shown in previous research to also contribute to overweight and obesity (Goryakin et al., 2015, Vogli et al., 2014). The index is based on the conceptualisation by Keohane and Nye (2000) who proposed three distinct dimensions of globalization: (1) economic: long distance flows of goods, capital and services as well as information and perceptions that accompany market exchanges, (2) political: the diffusion of government policies internationally, and (3) social: the spread of ideas, information, images, and people. For all dimensions, this index was created using comprehensive data collected annually since 1970. In our main analysis we use the overall KOF globalization index. The KOF globalization index, including its three subcomponents, has been obtained from the KOF project website http://globalization.kof.ethz.ch, and is described in detail in (Dreher, 2006a).

We also included several control variables from the World Bank Development Indicators. Specifically, as soft drink sales were also found to increase with national per capita income (Basu et al., 2013), and as income is a determinant of health (Grossman, 2000), we also control for the logarithm of GDP per capita in all our models (in constant, 2005 US dollars). We also control for other potential correlates of soft drink sales and BMI – the proportion of the population living in urban areas, proportion of the population aged 15–64, and the proportion of women in the population, using data from the World Bank indicators. As soft drink and fast food consumption can go hand in hand, the latter may confound the association of the former with overweight/diabetes. Although we do not have a reliable measure for fast food sales, we expect that controlling for globalization and urbanization – both potentially important determinants of fast food consumption (Mendez and Popkin, 2004) – would tend to alleviate this concern. In addition, as both soft drink prices and BMI/obesity may follow a time trend, we also control for time dummies.

Finally, using year-specific thresholds applied to GDP per capita, current US$ (Atlas method) from the World Bank, we split countries into two groups: low and lower middle income countries (which we abbreviate, for convenience, as LMICs) vs upper middle and high income countries (UHICs). As the income classification does vary over the observation period for some countries, we used the 2013 year-specific income group definition, and applied it to each country over the whole period. Using this classification, the data included 63 LMICs and 15 UHICs (see Annex).

### 2.2 Analysis

We estimate our associations of interest using ordinary least squares (OLS) multivariate regressions models as a baseline specification. As described below, we further take advantage of the longitudinal nature of the data by controlling for time effects, as well as for country-level fixed effects.

When estimating the associations between soft drink *prices* and BMI/weight/diabetes, it is important to control for cross-country differentials in the local price levels. We deal with this by using standardized, US$-denominated prices in all countries, adjusted by the PLI. Furthermore, any potential association between the variables of interest may be confounded by heterogeneity, both time-varying and time-invariant. We deal with the latter by controlling for country fixed effects (CFE), which may proxy for the potential determinants of both weight/diabetes and price levels, which do not change over time. Conditional on the assumption that the residual error term is uncorrelated with the soft drink sales/prices after controlling for CFE and other variables of interest, the CFE estimator is unbiased. However, using CFE comes at a cost of a less precise estimation than under the alternative random effects assumption (Cameron and Trivedi, 2005). Nevertheless, as the random effects assumption is more restrictive than the fixed effects one, we prefer to be more conservative in our estimation approach, and consistently control for CFE across all specifications (besides the baseline OLS estimates). Formally, we aim to estimate parameters in the following equation:

(1) $${\text{Y}}_{\text{it}}^{\text{j}}=\alpha +\beta_{1}X_{\mathrm{it}-1}+Z_{\mathrm{it}}^{\prime }\beta_{2}+\alpha_{i}+\varepsilon_{\text{it}}$$

where ${\text{Y}}_{\text{it}}^{\text{j}}$ is one of the four outcome variables *j* associated with country *i* at time *t*; X_it-1_ captures lagged soft drink sales per capita, or PLI-adjusted price; **Z_it_** is the vector of control variables as described above, with the associated parameter vector β_2_; *α_i_* are country fixed effects, possibly correlated with X and **Z**, and *ɛ_it_* is an error term.

Another complication is that health behaviours may cluster. For example, lack of physical exercise (Hill et al., 2003), soft drink and fast food sales can correlate with each other (Malik et al., 2006). Again we assume that controlling for country and time effects, as well as for as globalization and urbanization (both of which are potentially important drivers of these health behaviours and of health in general (Goryakin et al., 2015, Goryakin and Suhrcke, 2014)), should help alleviate this concern. However, we cannot rule out that some important variables may be omitted. To deal with this issue, we will perform a simple falsification check, based on the assumption that bottled water sales per capita, as well as the price of the bottled water, should be unrelated to any of our outcome variables. If this is not the case, then there can be some confounding mechanism common to both soft drink and bottled water equations. For example, propensity to exercise, to consume fast food, or some socioeconomic dimension that we are unable to control for, may be correlated with both soft drink and bottled water sales. Any potential correlation that we find between bottled water sales/prices and our outcome variables of interest may reflect this residual confounding, which might also apply in the case of the soft drink sales/prices equations.

## 3 Results

### 3.1 Descriptive statistics

Table 1 presents basic descriptive statistics, showing that between 1999 and 2014, the average PLI-adjusted price of soft drinks has been decreasing for the countries in this study. Compatible with this trend, soft drink sales per capita has been on the increase around the world, which is in line with previous evidence (Basu et al., 2013). These trends have been accompanied by a small but steady increase in mean BMI, as well as by much more marked increase in average overweight, obesity and diabetes prevalence in our global sample of countries.

**Table 1:** Descriptive statistics.

Year	Price for soft drinks, PLI-adjusted	Annual soft drink sales per capita, L	BMI, kg/m2	Overweight, %	Obese, %	Diabetes, %
1999	4.05	23.94	23.26	29.65	8.14	6.66
2000	3.98	24.33	23.33	30.19	8.39	6.80
2001	4.04	24.74	23.39	30.74	8.64	6.93
2002	3.91	24.71	23.46	31.30	8.90	7.07
2003	3.54	25.02	23.53	31.88	9.17	7.20
2004	3.29	25.46	23.61	32.49	9.47	7.34
2005	3.21	25.71	23.68	33.11	9.77	7.48
2006	2.99	26.10	23.75	33.76	10.10	7.61
2007	2.66	26.24	23.83	34.43	10.44	7.75
2008	2.40	26.14	23.91	35.11	10.79	7.88
2009	2.41	26.06	23.98	35.76	11.13	8.00
2010	2.06	26.25	24.05	36.43	11.49	8.13
2011	1.93	26.23	24.12	37.10	11.85	8.26
2012	1.87	26.25	24.18	37.76	12.23	8.38
2013	1.89	26.01	24.24	38.43	12.61	8.51
2014	1.90	25.94	24.30	39.08	12.99	8.65

*Source*: Euromonitor (2014), and Global Burden of Metabolic Risk Factors of Chronic Diseases data (downloaded in 2015). Annual average estimates are weighted by the country population. Standard errors are clustered on a country level.

**Fig. 1:**
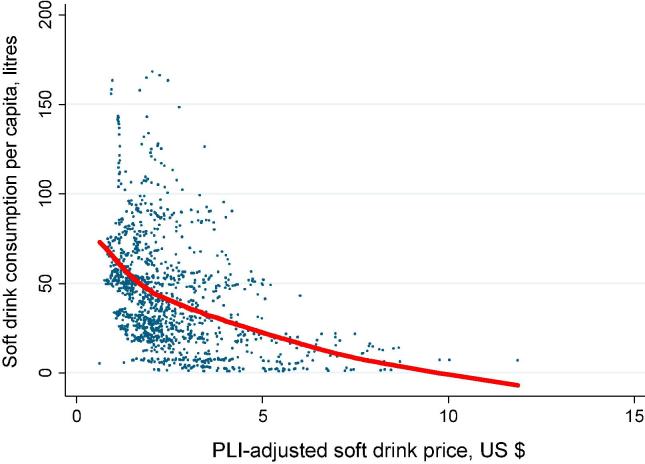
Lowess curve of the relationship between prices and soft drink sales (1999–2014). *Source*: Euromonitor (2014). *Note*: each dot represents one country in one year.

**Fig. 2:**
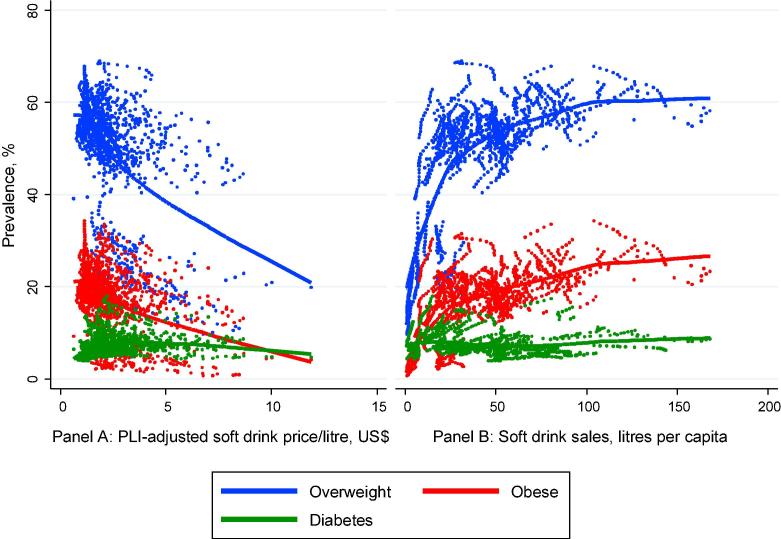
Lowess curve of the relationship between relative soft drink prices and mean overweight/obesity/diabetes prevalence (Panel A), and between per capita soft drink sales and mean overweight/obesity/diabetes prevalence (Panel B), 1999–2014. *Source*: Euromonitor (2014), and Global Burden of Metabolic Risk Factors of Chronic Diseases data (downloaded in 2015). *Note*: each dot represents one country in one year.

Fig. 1, Fig. 2 below provide a first glimpse of the bivariate relationships between the main variables of interest, using locally weighted scatterplot smoothing (‘lowess’) graphs.

Fig. 1 indicates that, as expected, PLI-adjusted soft drink prices are quite strongly negatively related to soft drink sales. Fig. 2 similarly suggests that soft drink prices are strongly negatively related to mean overweight and obesity prevalence. Likewise, there is a clear positive relationship, if at a decreasing rate, between soft drink sales per capita and average overweight and obesity prevalence. However, the relationship is not pronounced when diabetes is used as the outcome variable.

This simple preliminary analysis seems to support prior expectations regarding the direction of the relationship between the main variables of interest. However, as it might be driven by omitted covariates and time trends, we consider these issues further in the next section.

### 3.2 Regression analysis

#### 3.2.1 Do higher soft drink prices reduce soft drink sales?

We start by estimating the elasticity of soft drink sales with respect to their relative prices. In Table 2 (and in all tables that follow), we only present the main parameters of interest. For example, in column 1, the parameter of −0.209 was obtained from regressing log soft drink sales on log relative prices for soft drinks (lagged by 1 year), controlling for the percentage living in urban areas, the percentage aged 15–64 years, the proportion of females (all out of the total population), log GDP per capita, a measure of the country’s degree of globalization, regional dummies and time effects.

**Table 2:** Price elasticities of soft drink sales.

	(1)	(2)	(3)	(4)
	All countries	All countries	UHICs	LMICs
PLI-adjusted price, soft drinks	−0.209	−0.087^*^	−0.321^***^	−0.066
	(0.132)	(0.046)	(0.055)	(0.055)
Country fixed effects	No	Yes	Yes	Yes
Observations	1162	1162	225	937
R-squared	0.766	0.515	0.709	0.505

Outcome variables and relative prices are in log form. All models control for% living in urban areas,% aged between 15and 64; proportion of females in total population, log GDP per capita, total globalization index, regional dummies, time effects. Relative price is lagged 1 year. UHICs: high income and upper middle income countries; LMICs: low, lower middle and middle income countries. Standard errors are clustered on a country level.

As was the case in Fig. 1, the bivariate association in the preferred CFE model is negative. The relationship appears to be much more pronounced in the sample of the UHIC countries, where each 1% increase in the PLI-adjusted price of soft drinks is related to about 0.3% fall in soft drink sales per capita.

#### 3.2.2 Do higher soft drink sales increase mean BMI, as well as overweight and obesity prevalence?

Our baseline estimates suggest that when all countries are pooled together, each litre increase in per capita sales of soft drinks is associated with a 0.02 unit increase in BMI in the OLS specification (Table 3).

**Table 3:** Associations of soft drink sales per capita with weight-related outcomes.

	(1)	(2)	(3)
	BMI	Overweight%	Obese%
	OLS models		
Soft drink sales, per capita	0.019***	0.116***	0.098***
	(0.005)	(0.044)	(0.024)
Observations	1162	1162	1162
R-squared	0.740	0.829	0.795
	Country fixed effects included		
Soft drink sales, per capita	0.001	-0.002	0.006
	(0.002)	(0.007)	(0.012)
Observations	1162	1162	1162
R-squared	0.912	0.962	0.903

^**^ p < 0.05, ^*^ p < 0.1

Source: NCD Risk Factor Collaboration dataset. All models control for% living in urban areas, % aged 15–64, proportion of females in total population, log GDP per capita, total globalization index, regional dummies, time effects. Soft drink sales per capita are lagged 1 year. Standard errors are clustered on a country level.

*** p < 0.01

Likewise, the same increase in sales implies a significant increase in the risk of obesity by 0.10 percentage points (p.p.), and of overweight by 0.116 p.p. Nevertheless, these findings are not robust to controlling for country fixed effects in the longitudinal dataset, as all parameters turn insignificant.

Next, in Table 4 we estimate differential associations between soft drink sales and weight outcomes among low and lower-middle income countries vs upper-middle and high income countries, in all cases controlling for country fixed effects. The association is now positive and statistically significant in the LMICs subsample, and it is also robust to the use of the alternative control set. Specifically, in that set, we control for the separate components of the globalization index, as well as for three additional variables from the Food and Agricultural Organisation (FAO) proxying for the energy availability in a given country – food, protein and fat supply per capita.^4^ The falsification check also confirms that bottled water sales per capita are unrelated to any of our weight-related outcome variables of interest. Finally, the association is insignificant in the UHICs subsample.

**Table 4:** Estimation of the associations of soft drink sales per capita with weight-related outcomes, by income level, based on country fixed effects models.

	(1)	(2)	(3)
	BMI	Overweight%	Obese%
	LMICs		
Soft drink sales, per capita	0.009***	0.022*	0.059***
	(0.002)	(0.012)	(0.018)
Observations	225	225	225
R-squared	0.966	0.978	0.904
	LMICs, alternative controls*		
Soft drink sales, per capita	0.009***	0.044**	0.085**
	(0.003)	(0.018)	(0.031)
Observations	176	176	176
R-squared	0.978	0.982	0.918
	LMICs		
Bottled water sales, per capita	0.001	0.018	0.014
	(0.002)	(0.011)	(0.016)
Observations	225	225	225
R-squared	0.958	0.979	0.888
	UHICs		
Soft drink sales, per capita	-0.000	-0.007	-0.003
	(0.002)	(0.008)	(0.012)
Observations	937	937	937
R-squared	0.906	0.963	0.928

Source: Risk Factor Collaboration dataset. UHICs: high income and upper middle income countries; LMICs: low and lower middle countries. All models control for% living in urban areas,% aged 15–64, proportion of females in total population, log GDP per capita, total globalization index, regional dummies, country and time effects. Soft drink sales per capita are lagged 1 year. *The alternative controls set is:% living in urban areas,% aged 15–64, proportion of females in total population, log GDP per capita, economic, social and political globalization; food supply, kcal/capita/day; fat supply, g/capita/day; protein supply, g/capita/day; regional dummies, country and time effects. Standard errors are clustered on a country level.

*** p < 0.01.

** p < 0.05.

* p < 0.1.

Next, we find that soft drink sales are unrelated to diabetes prevalence in all specifications (Table 5).

**Table 5:** Association between soft drink sales on diabetes prevalence.

	(1)	(2)	(3)	(4)
	OLS	CFE	LMICs	UHICs
Soft drink sales, per capita	0.009	0.002	0.012	0.002
	(0.006)	(0.004)	(0.017)	(0.004)
Country fixed effects	No	Yes	Yes	Yes
Observations	1162	1162	225	937
R-squared	0.702	0.737	0.786	0.782

^***^ p < 0.01, ^**^ p < 0.05, ^*^ p < 0.1

Notes: Risk Factor Collaboration dataset. UHICs: high income and upper middle income countries; LMICs: low and lower middle countries. All models control for% living in urban areas,% aged 15–64, proportion of females in total population, log GDP per capita, total globalization index, regional dummies, country and time effects. Soft drink sales per capita are lagged 1 year. Standard errors are clustered on a country level.

In Table 6, the association between relative prices and all four outcomes of interest is shown. First, consider the top part of the table. As expected, BMI and overweight are negatively related to increases in the PLI-adjusted price of soft drinks, both in the pooled sample and in the sample of UHICs. Thus, in the overall sample, each one point increase in the price of soft drinks is related to decreases in BMI by about 0.03 units, and in the risk of overweight by 0.17 p.p. This association becomes much more pronounced in the sample of UHICs, including for obesity and diabetes.

**Table 6:** Estimation of the association between soft drink prices and weight-related outcomes and diabetes, using different price definitions, based on country fixed effects models.

	(1)	(2)	(3)	(4)	(5)	(6)	(7)	(8)	(9)	(10)	(11)	(12)
	BMI	Overweight%	Obese%	Diabetes%	BMI	Overweight%	Obese%	Diabetes%	BMI	Overweight%	Obese%	Diabetes%
	Pooled sample				LMICs				UHICs			
SD price, PLI-adjusted	-0.025^**^	-0.166^**^	-0.041	-0.074	0.004	0.079	0.167	0.016	-0.033^*^	-0.251^**^	-0.150^*^	-0.132^**^
	(0.012)	(0.075)	(0.081)	(0.053)	(0.020)	(0.106)	(0.135)	(0.112)	(0.016)	(0.095)	(0.086)	(0.052)
Observations	1162	1162	1162	1162	225	225	225	225	937	937	937	937
R-squared	0.915	0.964	0.903	0.742	0.958	0.978	0.889	0.783	0.909	0.965	0.930	0.798
SD relative to bottled	0.003	-0.113	-0.267^**^	0.038	0.043	0.256	0.203	0.172	-0.041^**^	-0.317^***^	-0.322^***^	0.032
Water price	(0.015)	(0.122)	(0.121)	(0.044)	(0.039)	(0.173)	(0.234)	(0.115)	(0.017)	(0.092)	(0.101)	(0.053)
Observations	1162	1162	1162	1162	225	225	225	225	937	937	937	937
R-squared	0.912	0.963	0.907	0.737	0.960	0.979	0.888	0.790	0.908	0.964	0.931	0.782
SD relative to wheat price	-0.004	-0.053^**^	-0.050^**^	0.010	0.021	0.123^*^	0.114	-0.001	-0.007^*^	-0.066^***^	-0.052^**^	-0.001
	(0.004)	(0.023)	(0.023)	(0.012)	(0.014)	(0.062)	(0.082)	(0.029)	(0.004)	(0.024)	(0.024)	(0.012)
Observations	870	870	870	870	114	114	114	114	756	756	756	756
R-squared	0.919	0.965	0.937	0.764	0.964	0.983	0.932	0.918	0.919	0.966	0.946	0.786
Bottled water price, PLI-adjusted	-0.046^***^	-0.300^***^	-0.256^**^	-0.15^***^	-0.043	0.027	0.028	-0.054	-0.04^***^	-0.311^***^	-0.253^***^	-0.151^***^
	(0.014)	(0.089)	(0.104)	(0.055)	(0.041)	(0.180)	(0.238)	(0.148)	(0.014)	(0.097)	(0.081)	(0.045)
Observations	1162	1162	1162	1162	225	225	225	225	937	937	937	937
R-squared	0.917	0.965	0.907	0.750	0.960	0.977	0.885	0.784	0.910	0.966	0.932	0.799

Notes: Risk Factor Collaboration dataset. UHICs: high income and upper middle income countries; LMICs: low and lower middle countries. All models control for % living in urban areas, % aged 15–64, proportion of females in total population, log GDP per capita, total globalization index, regional dummies, country and time effects. Soft drink sales per capita are lagged 1 year. Standard errors are clustered on a country level. PLI: price level index.

Next, we test the robustness of these findings by using a different definition for the relative soft drink prices. First, we divide the soft drink price per litre by the bottled water price per litre, in each country. This price metric is useful for assessing how relatively less expensive the alternative of consuming non-sugary drinks is compared to soda. In accordance with basic microeconomic theory, we assume that the more expensive the soft drinks are relative to bottled water, the less likely people will be to choose the former. One might reasonably object that local characteristics such as the availability of fresh, potable water may drive the pricing of bottled water (as well as whether people choose to drink tap water instead). We account for this by allowing for country-specific fixed effects. Again, our results suggest that price is strongly negatively related to weight-related outcomes in the sample of UHICs, implying that for each doubling of the price of soft drinks relative to bottled water, there is a drop of overweight and obesity prevalence by about 0.3 p.p.

Finally, we adjust soft drink prices by the prices of wheat per ton.^5^ The magnitude of the parameters is not directly comparable with the other two sets discussed in this section, but it is instructive that an increase in the price of soft drinks relative to the price of wheat in a given country is again negatively related to the decrease in BMI, as well as in overweight/obesity/diabetes prevalence, especially in the sample of UHICs. Results in Table 5 also indicate that diabetes is mostly unrelated to the variation in prices across specifications with different price definitions.

Although the above results strongly suggest that soft drink prices are negatively related to weight-related outcomes in the UHICs sample, we still need to confirm with the falsification check that the PLI-adjusted price of the bottled water is not significantly related to these outcomes. As it turns out, however, the bottom part of Table 5 shows that bottled water prices are strongly negatively related to the outcome variables both in the pooled and in the UHICs sample, implying that our soft drink price models may suffer from potentially important unobserved confounding.

In the above estimates we assumed that soft drink sales/prices affect BMI/diabetes with a one-year lag. One might object to this on the grounds that a cumulative condition such as BMI or diabetes can be driven through other lags of prices and sales as well. However, a priori it is not obvious what the appropriate lag structure should be. One way to statistically test the impact of accumulated lags is with the help of the Koyck distributed lag model (Wooldridge, 2015), which enables estimation of the accumulated effect of the variable of interest, under certain assumptions. We discuss this approach further in the Annex.

## 4 Policy implications

To date, the evidence on the impact of soft drink sales on overweight/obesity has been dominated by studies from North America which may have little applicability to other contexts (Gibson, 2008). The present paper adds to the evidence base by helping fill several gaps in the existing literature. For example, there is a dearth of studies using more advanced methods of controlling for unobserved heterogeneity (e.g. via country fixed effect estimation), or which consider additional metabolic disorder-related outcomes, such as diabetes. There are also very few studies which consider the association between prices of soft drinks and these outcome variables, in particular for low and middle income countries.

We found that in the sample containing all countries, soft drink sales were positively related to BMI, obesity and diabetes in the baseline OLS models, which was also consistent with findings from several recent systematic reviews (Imamura et al., 2015, Pereira, 2006, Vartanian et al., 2007). Nevertheless, this relationship was rendered insignificant after controlling for country fixed effects, implying that unobserved time-invariant heterogeneity accounts for a significant part of the positive association observed in the OLS models. It is unclear a priori why controlling for CFE makes such a large difference, but it might be the case that some local preferences for sugary or generally unhealthy foods may introduce positive bias if this relationship is estimated by OLS. This was not the case in the LMICs sample, as the association between per capita soft drink sales and all three weight-related outcomes continued to remain significant even after controlling for country fixed effects, as well as after running several robustness and falsification checks.

Nevertheless, even in the LMICs sample, the magnitude of this association was modest, with each litre increase in soft drink sales per capita per year leading only to a 0.009 unit increase in BMI in the more robust CFE model (Table 4).This translates to a 0.26 greater BMI for one standard deviation drop in soft drink sales, which would explain about 16% of one standard deviation of the BMI distribution. Another way to look at it is that annual soft drink sales range from 0.9 to 70 litres per capita in the LMICs. Reducing sales from the highest to the lowest level in that group of countries could potentially lead to a reduction of BMI by about 0.62 kg/m2, or by about 3%. More informatively, however, would be to consider the effect of changes in the annual soft drink consumption in LMICs, which is about 6 litres per capita. A very ambitious goal of halving this level would be predicted to lead to only about a 0.03 kg/m^2^ reduction in BMI, a 0.06 p.p. reduction in overweight, and a 0.18 p.p. reduction in obesity prevalence.

Another ecological study which used similar data yet with a different focus (De Vogli et al., 2014) found that soft drink sales per capita were mediating the relationship between fast food sales and age-standardized BMI, although the parameter on soft drink sales was not significant in the model which adjusted for potential confounders, including the log of per capita GDP. Our study does, however, differ in several respects: first, in the De Vogli et al. paper, the main association of interest was between fast food sales and BMI, while the role of soft drink sales was of secondary interest. Also, De Vogli et al. (2014) did not consider the effect of soft drinks on diabetes, and neither the role of prices. Our sample of countries is also considerably larger (n = 78) and more globally representative compared to their dataset, which was restricted to 25 OECD countries.

Several policy options can potentially be used to reduce soft drink consumption, ranging from taxation of soft drinks, to advertising and sales restrictions, and to the regulation of product labelling. For example, there is evidence that pre-packaged food labelling (and in particular contextual and interpretive types) is effective at prompting people to choose healthier foods (Cecchini and Warin, 2016). The labelling for sodas can potentially be even more effective if goes beyond being purely informative, by providing a clear warning about the health risks associated with excessive sugar consumption (Cohen and Lesser, 2016). Limits can also be placed at a point of purchase, as when 16 oz single serving limit for soft drinks was imposed in New York city (Cohen and Lesser, 2016), or when France introduced outright bans on unlimited soda drinks offers.

The evidence on the effectiveness of these options is still limited. While it indeed appears that soda (and, more broadly, unhealthy food) consumption may decrease in response to higher prices (Bíró, 2015, Wada et al., 2015), the evidence is weaker for weight-related outcomes. For example, Fletcher et al. (2010) found that in the US, there was a small negative effect of state-level soft drink taxes on BMI, as well as on obesity and overweight, with one percentage point increase in taxes being associated with a decrease in BMI of about 0.003. Given that the average tax rate at the time of the study was about 3% in the US, the magnitude of the effect appears quite small. Likewise, Kim and Kawachi (2006) found no association between obesity prevalence and state soft drink or junk food taxes in the USA, while Schroeter et al. (2008) found that a 10% soft drink tax increase in the US would be associated with a decrease of body weight by only 0.1%. Our initial finding of a small negative association of prices with weight-related outcomes in UHICs is again consistent with the previous literature, but the fact that bottled water prices were also strongly negatively related to the same outcomes suggests that some residual confounding may be driving both relationships.

In interpreting our findings, one needs to bear in mind several limitations of our approach. First, using country-level data may be a somewhat blunt instrument for estimating own price elasticities, especially given that prices and sales may be simultaneously determined. A more appropriate approach would be estimating a demand system, where prices are instrumented with exogenous drivers such as tax levels. Unfortunately, the lack of data on soft drink taxes for all countries in our sample makes such an estimation infeasible. Alternatively, more significant estimates could potentially be found with individual or household-level data on the consumption of soft drinks, linked with regional or time series data on soft drink prices. One systematic review which considered household and individual level evidence on price elasticity of demand for SSBs did find price elasticity of demand for carbonated soft drinks to be -1.25 and significant (Powell et al., 2013). In another recent study, a 10% increase in soda prices was associated with 4% lower prevalence of regular soda intake among adults (Wada et al., 2015).

In addition, our outcome variables do not always reflect empirically observed data, but instead are often estimates, if carefully derived ones, based on a large number of surveys, and on related direct and indirect epidemiological evidence (Danaei et al., 2011a, Danaei et al., 2011b, Finucane et al., 2011b), thereby introducing potentially important non-random measurement error. In addition, there is no distinction between sugary and diet soft drinks (as well as their prices), and we cannot exclude the possibility that – had our data on carbonated soft drinks been made up exclusively of sugar-sweetened beverages, the association might well have been stronger (Basu et al., 2013). That said, there is also some evidence that diet soft drinks may promote weight gain (Azad et al., 2017, Fowler et al., 2015), even though the evidence base here is still emerging, and the policy focus has thus far not been on this soft drink sub-category. Also, soft drink sales data are industry estimates derived from consumer retail off-trade purchasing data (Basu et al., 2013), and as such may also suffer from a degree of measurement error.

Moreover, overall sales may be an imperfect proxy for consumption among adults, as there can be considerable waste, as well as sales among children, which may also bias downward our parameter estimates on sales. The impact of sales/prices may also operate with a considerable lag, which was not possible to capture, given the restricted period for which data was available. Finally, even though we have an equal split between LMICs and HICs, the relatively small number of countries in each group and overall (78) implies that our results may not be fully representative for all countries in the world.

## 5 Conclusion

Overall, although we did find some evidence that soft drink sales are a statistically significant predictor of BMI and obesity, at least in the sample of low and lower middle income countries, the magnitude of this effect was small. However, this does not imply that soft drink sales and prices are an insignificant driver of obesity, but it does highlight the potential limitations of using ecological research design when studying this association. Therefore, results from individual level studies will be required to further ascertain the role of prices and of soft drink sales in obesity and diabetes.

## Footnotes

1 This project is coordinated by the WHO Collaborating Centre on NCD Surveillance and Epidemiology at Imperial College London (http://ncdrisc.org/about-us.html)

2 For simplicity, we will use “soft drinks” and “carbonated soft drinks” terms interchangeably

3 https://blogs.worldbank.org/opendata/category/tags/icp

4 http://www.fao.org/faostat/en/

5 The data is from FAOSTAT: http://www.fao.org/faostat/en/#data
