# Supplementary material for: Soft drink prices, sales, body mass index and diabetes: Evidence from a panel of low-, middle- and high-income countries
Source: Food Policy. 2017 Dec;73:88–94. doi: 10.1016/j.foodpol.2017.09.002 (PMC5727680; doi:10.1016/j.foodpol.2017.09.002)
Supplement: Supplementary data 1 [file mmc1.docx]

**Annex**

**Table A1**.Countries in the analytical sample

| **High income and upper middle income countries** | **Low, and lower middle income countries** |
| --- | --- |
| Algeria | Bolivia |
| Argentina | Cameroon |
| Australia | Egypt |
| Austria | Georgia |
| Azerbaijan | Guatemala |
| Belarus | India |
| Belgium | Indonesia |
| Bosnia and Herzegovina | Kenya |
| Brazil | Morocco |
| Bulgaria | Nigeria |
| Canada | Pakistan |
| Chile | Philippines |
| China | Ukraine |
| Colombia | Uzbekistan |
| Costa Rica | Vietnam |
| Croatia |  |
| Czech Republic |  |
| Denmark |  |
| Dominican Republic |  |
| Ecuador |  |
| Estonia |  |
| Finland |  |
| France |  |
| Germany |  |
| Greece |  |
| Hungary |  |
| Iran |  |
| Ireland |  |
| Israel |  |
| Italy |  |
| Japan |  |
| Kazakhstan |  |
| Korea |  |
| Latvia |  |
| Lithuania |  |
| Macedonia |  |
| Malaysia |  |
| Mexico |  |
| Netherlands |  |
| New Zealand |  |
| Norway |  |
| Peru |  |
| Poland |  |
| Portugal |  |
| Romania |  |
| Russian Federation |  |
| Saudi Arabia |  |
| Serbia |  |
| Singapore |  |
| Slovakia |  |
| Slovenia |  |
| South Africa |  |
| Spain |  |
| Sweden |  |
| Switzerland |  |
| Thailand |  |
| Tunisia |  |
| Turkey |  |
| United Arab Emirates |  |
| United Kingdom |  |
| United States |  |
| Uruguay |  |
| Venezuela |  |

A2. Koyck distributed lag model.

Although the Koyck distributed model is generally estimated for pure time series data, there are also examples in the literature, e.g. ([Brown, 2014](#_ENREF_1)), that also estimated this model using panel data. The required assumption for this approach is that soft drink sales/prices have a geometrically declining impact on BMI/diabetes over time, which seems reasonable enough. The cumulative impact estimated with the help of the model will be the sum of all these impacts going back infinitely into the past. After some arithmetical manipulations (the details can be found e.g. in ([Brown, 2014](#_ENREF_1); [Wooldridge, 2015](#_ENREF_3)), the model to be estimated is as follows:

Y_ct_ =βX_ct_+γY_ct-1_+ α_i_ + ε_it_

After arithmetic manipulations, the following formula represents the so-called long-run propensity (LRP), which in effect is the cumulative impact of a permanent increase (measured over multiple years) in soft drink sales/prices (we estimate standard errors with the delta method):

LRP= β/(1-γ)

The short run propensity is measured by the parameter β.

The complication here is that including a lagged dependent variable in the model with serially correlated errors (which will be the case after eliminating fixed effect α_i_) will lead to inconsistent parameter estimates. We deal with this issue by estimating the Arellano-Bond model ([Cameron and Trivedi, 2009](#_ENREF_2)). In short, we first eliminate the fixed effect α_i_ with first differencing, and then apply instrumental variable estimation of the parameter γ, using lags of repressors as instruments. Of course, this approach will only work if the instruments are sufficiently strongly correlated with the repressors of interest. In addition, the Arellano-Bond model requires the testable assumption that the errors in the first-differenced model are serially uncorrelated (we test this using estat abond command for 3 lags^[[1]](#footnote-1)^). We try to relax it by including an additional lag of the dependent variable in our specifications.

The results are presented in Table A2 (for simplicity, we only show estimates for the long-run propensity). However, given the evidence that the errors are still serially correlated in all models (implying model misspecification), we should not over-interpret these findings.

**Table A2**. Arellano-Bond estimation

|  | BMI |
| --- | --- |
| LRP, Soft drink sales, per capita | 0.001 |
|  | (0.001) |
| Observations | 1162 |
| Number of countries | 78 |
| Evidence of misspecification? | Yes |
|  | diabetes |
|  |  |
| LRP, Soft drink sales, per capita | -0.000 |
|  | (0.0001) |
| Observations | 1162 |
| Number of countries | 78 |
| Evidence of misspecification? | Yes |

Notes: All models control for % living in urban areas, % aged 15-64 proportion of females in total population, log GDP per capita, total globalization index, regional dummies, time effects. Evidence of misspecification is provided using estat abond, artest(3) command.

References

Brown, T.T., 2014. How effective are public health departments at preventing mortality? Economics & Human Biology 13, 34-45.

Cameron, A.C., Trivedi, P.K., 2009. Microeconometrics using stata. Stata press College Station, TX.

Wooldridge, J.M., 2015. Introductory econometrics: A modern approach. Nelson Education.

1. If errors are serially uncorrelated, we expect to reject the null of zero autocorrelation in the first-differenced errors at order 1, but not at higher order ([Cameron and Trivedi, 2009](#_ENREF_2)). [↑](#footnote-ref-1)
